# English Hospitals through French Glasses

**Published:** 1905-09-09

**Authors:** 


### English Hospitals Through French Glasses.

The *entente cordiale* is having its anticipated effect in stimulating the mutual curiosity of the two allied nations. We in England have always been interested in the methods of our Gallic neighbours, and have on many points been ready to admit that "they manage these things better in France." What seems novel in the situation is the awakening of a similar interest across the Channel in the English way of doing things.

The *Revue de Deux Mondes* has recently published, under the title of "Une Visite aux Hospitaux de Londres," an entertaining description of the leading London hospitals. The thin veil of anonymity assumed by the author is supposed to conceal the personality of an eminent Frenchwoman. It is well to see ourselves as others see us, and not all our visitors have been so picturesque or so sympathetic in their accounts of us as this lady. At the same time, the most observant and intelligent tourist remains a tourist—that is to say, a skimmer of the surfaces of things—and more than once in reading this admirably written sketch we have been reminded of the learned German professor who recently assured his countrymen, in a serious work on the habits of our nation, that the typical young man of the English upper classes took a scent bath every morning.

The author of the article has visited the great metropolitan hospitals, beginning with the Royal Free, which she finds "as gay as a hospital in an opera. The beds are spread out, not more than sixty in the large room. Everything shines, the varnished wooden tables, the picture frames on the walls, the white caps of the pretty nurses, everything smiles with the smile of joyful convalescence. And that piano? Is it a fête? . . . There are flowers in bunches, in handfuls; their magic is everywhere . . .

"But death enters all the same. . . . Here, it is very near a child who has ceased to smile. The fever which holds him in its grip will not leave him, his lost gaze sees nothing. I look at the chart which hangs in front of the bed—broncho-pneumonia. And it was a frail little creature. The parents are on either side of the bed, and on the knees of the mother a pallid baby is gravely sucking his empty India-rubber teat. For we are in a suburb of London, and poverty is close at hand."

At St. George's Hospital the visitor notes the daily cost of a patient, "which attained last year 10.20 frs. (7s. 9d.) per day. At the Royal Free it does not go beyond 4.70 frs. These terms are the two extremes in the statistical ladder.

Naturally the opportunities offered to women for medical study have a special interest for this observer. She visits the School of Medicine at Hunter Street and the Hospital in the Gray's Inn Road, where she meets "Mrs. ——, a lady surgeon of great reputation, whose operating method has formed a school. She has a numerous *clientele*, and her gynæcological lectures at the Royal Free Hospital are assiduously followed." At the New hospital in the Euston Road she notes the excellence of the surgical treatment and the large number of out-patients.

She is particularly struck with the spirit of seriousness and devotion in which the young medical women pursue their careers, and she notes the fact that nearly one-fourth of the students from the London School of Medicine for Women have devoted themselves to mission work.

Guy's Hospital, St. Thomas's, and the London Hospital are passed in review in turn. One criticism of the writer's will cause some astonishment, not unmingled with amusement, to those who have practical experience of a nurse's life. She finds that they are made too comfortable. "The nurses are 'ladies,' *femmes du monde.* It would be ungracious to reproach them with having introduced certain exigencies," but the comfort of their surroundings "takes away from the profession some of its heroism."

Another point deals with the "religious difficulty." "Have you only Protestant nurses?" she asks the head of the Nursing Home at Plaistow. "Oh, no; all denominations are admitted. At the moment we have two Jews and several Roman Catholics." And the author exclaims, "Oh, what praiseworthy tolerance. Why have I not met it in the larger hospitals?"

Finally she draws an interesting comparison between the financial methods of charitable agencies in England and France.

"Plenty of money, plenty of generous benefactors, plenty of emulation. Tastes and habits of luxury and excessive comfort which one is tempted to display even outside one's own home. . . . I have given my money, I want to see the result. The more expensive the result appears the better satisfied I shall be. A hospital, if it wishes to receive money, must throw a good deal out of the window. It boasts of this and finds the system answer. 'You see that I have no more. Now, I must have more; so you will give it me.' This is the most peremptory of syllogisms and the Englishman gives. A system like this would run the risk of killing all our charities in France."

Those of our readers who understand French would find it interesting to turn to the article itself. However little we may agree with some of the observations or conclusions of the writer, this picture of our hospitals, taken from a point of view so different from ours, has at any rate the interest of novelty, and may not be altogether without instruction.

